# A Simple Analysis of the Second (Extra) Disulfide Bridge of V_H_Hs

**DOI:** 10.3390/molecules29204863

**Published:** 2024-10-14

**Authors:** Carla Martins, Fabrice Gardebien, Aravindan Arun Nadaradjane, Julien Diharce, Alexandre G. de Brevern

**Affiliations:** 1 Université Paris Cité and Université de la Réunion and Université des Antilles, INSERM, BIGR, DSIMB, F-75015 Paris, France; cmartins@insa-toulouse.fr; 2 Université Paris Cité and Université de la Réunion and Université des Antilles, INSERM, BIGR, DSIMB, F-97715 Saint Denis Messag, France; fabrice.gardebien@univ-reunion.fr (F.G.); aravindan.nadaradjane@univ-reunion.fr (A.A.N.)

**Keywords:** secondary structure, sequence structure relationship, structural alphabet, molecular dynamics, antibodies, frameworks

## Abstract

Camelids produce a special type of antibody, known as V_H_Hs, that has lost the V_L_ domain, providing a more optimised V_H_ domain. This particular highly stable antibody domain has interesting properties for biotechnological development. Ordinarily, those molecules possess only one disulphide bridge, but surprisingly, at least a quarter of these V_H_Hs have a second one. Curiously, this does not seem to be essential for the stability and the function of this domain. In an attempt to characterise precisely the role and impact of this disulphide bridge at the molecular level, several in silico mutants of a V_H_H were created to disrupt this second disulphide bridge and those systems were submitted to molecular dynamics simulation. The loss of the second disulphide bridge leads to an increase in the flexibility of CDR1 and CDR3 and an unexpected rigidification of CDR2. Local conformational analysis shows local differences in the observed protein conformations. However, in fact, there is no exploration of new conformations but a change in the equilibrium between the different observed conformations. This explains why the interaction of V_H_Hs is not really affected by the presence or absence of this second bridge, but their rigidity is slightly reduced. Therefore, the additional disulphide bridge does not seem to be an essential part of V_H_Hs function.

## 1. Introduction

**Figure 1:**
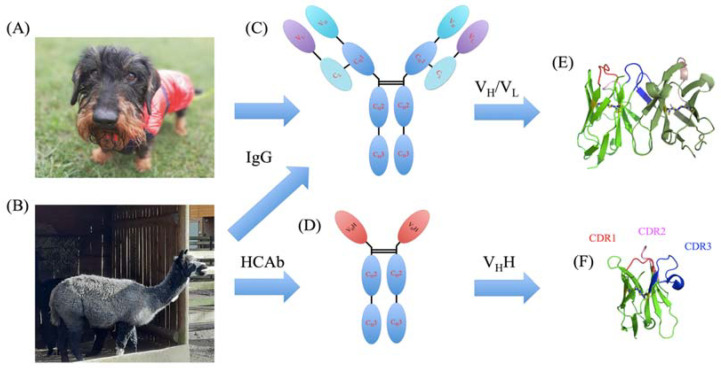
Comparison of V_H_/V_L_ and V_H_H domains. (**A**) A classical wirehaired dachshund named Snoopy that has classical IgGs and (**B**) a *Vicugna pacos* from Krakow Zoo that also has HCAbs. Schematic representation of (**C**) IgG and (**D**) HCAb with C_H_1, C_H_2, C_H_3, V_H_, C_L_, V_H_, and V_H_H domains with a 3D representation with CDR1 in red, CDR2 in pink, and CDR3 in blue colours for (**E**) V_H_/V_L_ and (**F**) V_H_H. Three-dimensional visualization was carried out with PyMOL [10,11,12].

Immunoglobulins (Ig) are large Y-shaped proteins used by the immune system to identify and neutralize antigens by direct interactions. They are composed of various different types of Igs, the most classical being IgG. Members of this family are composed of two heavy (with C_H_1, C_H_2, C_H_3, and V_H_ domains) and two light chains (with C_L_ and V_L_, domains, see Figure 1C,E). They are found in all mammals (see Figure 1A), but the members of the family *Camelidae* (including *Lama glama*, *Camelus bactrianus*, *Camelus dromedarius*, and *Vicugna pacos*) also have a supplementary modified variety of IgG which completely lacks (i) light chains (no C_L_ and V_L_ domains) and (ii) the C_H_1 domain (see Figure 1B) [1]. They were named heavy-chain-only antibodies (HCAb, see Figure 1D,F) [2,3] and they are important for camelid newborns [4]. In HCAbs, the deletion of the splicing consensus sequence in their mRNA results in the absence of the heavy chain C_H_1 domain [5,6]. In the absence of the V_L_ domain, the formerly hydrophobic V_H_-V_L_ interface has evolved to adapt to the hydrophilic environment [5,7]. The final VH domain of these HCAbs is called V_H_H (or nanobody [8]) [9].

V_H_, V_L_, and V_H_H domains are composed of four framework regions (FRs), highly conserved; they form a classic scaffold composed mainly of β-sheets. Three Complementary Determinant Regions (CDRs) link them; those regions are highly variable in terms of sequence as they provide the specificity that allows for the recognition of the epitope by the paratope. V_H_H domains are half the volume of the V_H_-V_L_ complex. The loss of binding surface area is compensated by the increase in CDR length (particularly CDR3); thus, they provide certain particular conformations less present in IgGs [13]. V_H_Hs have interesting experimental properties because they are easier to over-express than classical IgGs, e.g., in heterologous expression systems as *E. coli*, plant, or by phage display [14,15,16,17]. They have high thermal tolerance [18,19], induced by intramolecular interactions between the CDR3 and the FR2 region [20]. Their small size, ease of expression, and unique biochemical and biophysical properties have made V_H_H the harbingers of biotechnological tools used in healthcare therapeutics and diagnostics. Several innovative V_H_H drugs have been developed, e.g., Caplacizumab^®^ against acquired Thrombotic Thrombocytopenic Purpura [21,22,23], Ozoralizumab (Nanozora^®^) against rheumatoid arthritis [24,25], or Ciltacabtagene autoleucel (CARVYKTI^®^) against refractory/relapsed multiple myeloma [26,27].

The disulfide bridges of immunoglobulins were characterized decades ago; they are numerous and are found mainly within each domain [28,29,30]. The domains (C_H_s, C_L_, V_L_, and V_H_) of IgGs most often have one characteristic and a conserved disulphide bridge [31]. These bridges are highly important in V_H_ and V_L_ domains [32]. Disulphide bridges are also found between heavy chains and between heavy and light chains [33]. Non-classical disulphide bond structures were first identified in IgG_4_ and later in IgG_2_ antibodies, but they remain highly rare [34].

**Figure 2:**
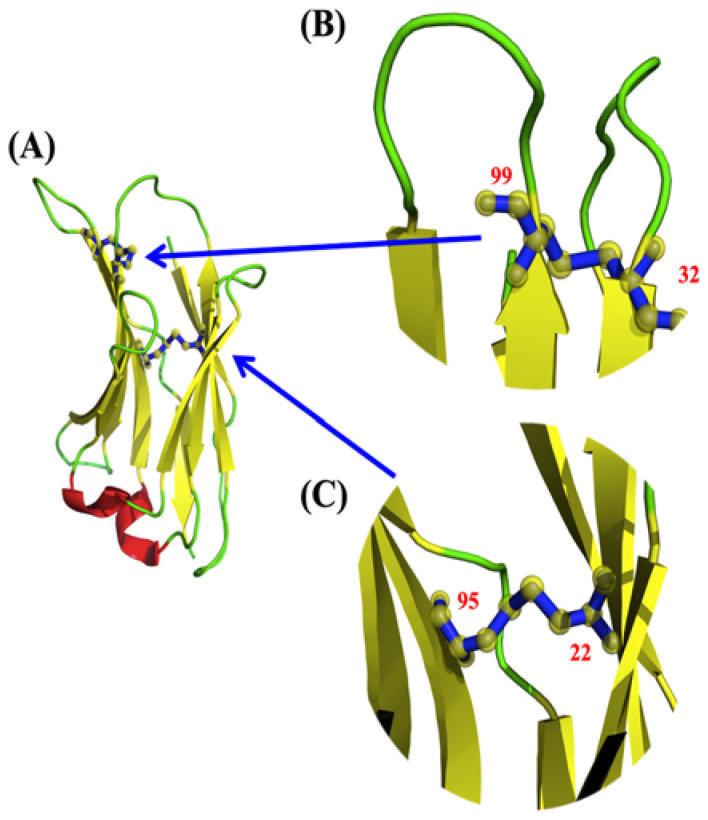
V_H_H disulphide bridges. (**A**) V_H_H PDB id 1YC7 [35], with the two disulphide bridges, (**B**) the supplementary disulphide bridge (positions 32 and 99), and (**C**) the canonical disulphide bridge (positions 22 and 95). Visualization was carried out with PyMOL [10,11,12].

Analysis of V_H_Hs has revealed the presence of other additional disulphide bridges not generally found in antibody V_H_ domains [35,36,37] (see Figure 2). Different types of additional disulphide bridges are observed in V_H_H sequences and almost always involve CDR1, more rarely FR2 or CDR2, and most commonly CDR3 [38,39,40]. However, the majority of these studies focus only on cysteine patterns, not disulphide bond evidence. We therefore draw your attention to an example that is representative of the majority of the Protein Data Bank’s V_H_Hs with an additional disulphide bond. It is found between CDR1 and CDR3 (IMGT positions C33–C102) [36,41]. Sequence and structure studies highlight that this second disulfide bridge, between CDR1 and CDR3, is found in a quarter of V_H_Hs [13,42,43]. It is the most common of the extra bridges; others are much rarer. Surprisingly, it seems that they are not as essential as could be expected [44,45,46].

Disulphide bridges are particularly interesting from a structural perspective and are often seen as essential for maintaining protein topology [47]. However, this is not always the case [48], raising specific questions in the context of these antibodies. An experimental study even showed that V_H_H with or without this second disulphide bridge had the same characteristics in terms of stability and affinity [44], while previous conclusions considered that its presence increases those properties [49].

Our research specifically investigates the existence of this not-so-rare additional disulphide bridge present between the hypervariable regions, CDRs. We therefore carried out in silico mutagenesis in four different experiments: (i) replacing the first Cysteine with an Alanine, (ii) then just the second Cysteine with an Alanine, (iii) then both Cysteines with Alanines, and finally (iv) by substituting Glycines for Cysteines. These five systems (wild types and four series of variants) are analysed comparatively by molecular dynamics simulations. Classical (RMSf) and innovative approaches (Protein Blocks [50] with PBxplore software [51]) are used. Our results shed light on the lack of role of this second disulphide bridge. In fact, its absence does not seem to affect their conformations and affects their dynamical behaviours only slightly. Although not intuitive at first, the results are consistent with the experimental results.

## 2. Results

### 2.1. Systems

**Figure 3:**
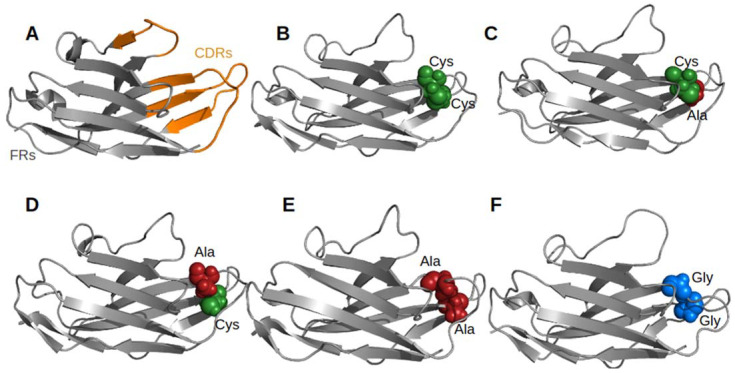
V_H_H structure and the 2nd disulphide bridge. (**A**) FRs (in grey) and CDRs (in orange) delimitation, (**B**) wild type, (**C**–**F**) mutants. In (**C**,**D**) single and (**E**,**F**) double mutants, Cysteines are in green balls, Alanines in red, and Glycines in blue.

While almost all V_H_H share a common canonical disulphide bond (found in this V_H_H at positions C23-C104), approximately only one-quarter have a second disulphide bond between CDR1 and CDR3 (found in this V_H_H at positions C32–C99). To elucidate its possible role in the antigenic binding of V_H_H by stabilizing the CDR3 loop, e.g., reducing the entropic penalty, a V_H_H that recognizes the surface glycoprotein carbohydrates of trypanosomes, parasites/hematophagous insects responsible for infections, the trypanosomiases (PDB ID 1YC7 [35], see Figure 3A,B) was selected. This V_H_H was selected as (i) it represents a classical length of CDRs of V_H_H and (ii) it was experimentally used as a concrete case for comparison with the V_H_ of V_H_-V_L_ complexes.

Single and double mutants have been built and examined to assess the effect of these mutations on the V_H_H structure by molecular dynamics simulations. Four mutants were produced: two single mutants (32A-C99 and C32-99A, see Figure 3C,D) and two double mutants (32A-99A and 32G-99G, see Figure 3E,F). The Glycine double mutant aims to apprehend the impact of aliphatic chains on the V_H_H structure.

### 2.2. Global Analyses

**Figure 4:**
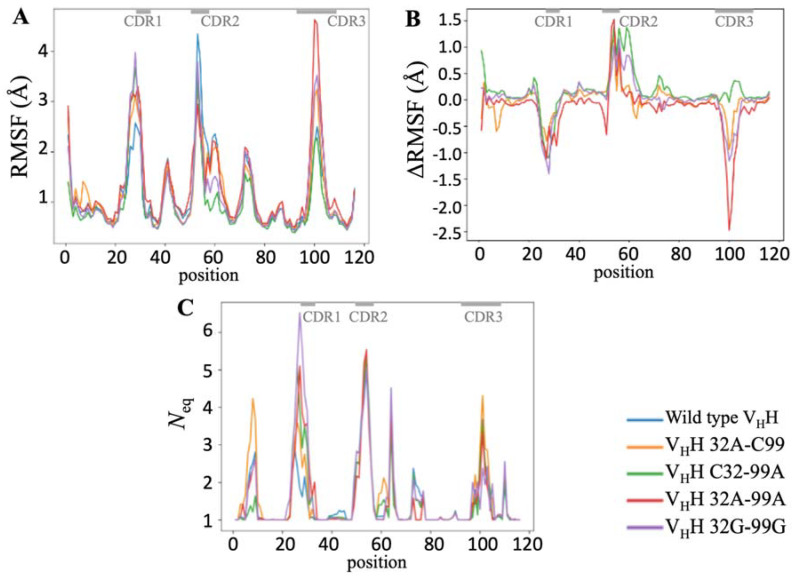
Changes in V_H_H without the second disulphide bridge. (**A**) RMSF, (**B**) ΔRMSF, and (**C**) *N*_eq_ (PB entropy). Colours are (**A**,**C**) wild-type V_H_H (blue), 32A-C99 (orange), C32-99A (green), 32A-99A (red), and 32G-99G (purple). The dashes at the top of each figure in grey represent the CDR positions.

For all conditions, the root mean square deviation increases and reaches a plateau around 1 and 2 Å; similarly, the repetitive structures of FRs remain also quite constant during all simulations. The structures remain stable, and so simulations can be analysed. Figure 4A shown RMSF values. High values are associated, as expected, with CDRs (approximately at positions 23–37 and 50–63—it also encompasses the beginning of FR3- and 99–105). An increase in flexibility is observed for all mutants in the CDR1 region (going from the highest value of 2Å to 4Å for C32-99A and 32G-99G). An increase in flexibility is also observed in the CDR3 region for 32A-99A (and not 32G-99G). Surprisingly, a decrease in flexibility (RMSF value) is found for all mutants on CDR2. ΔRMSF (see Figure 4B) analysis emphasizes those differences. The second disulfide bridge seems to have an impact on the flexibility of each CDR and not on FRs. Indeed, its presence brings a greater rigidity in the CDR1 and CDR3 loop and would lead to a greater flexibility in CDR2.

### 2.3. Local Analyses

A more detailed analysis of flexibility is carried out by the use of Protein Blocks [50]. The *N*_eq_ analysis (see Figure 4C) provides a more complex and finest analysis [51,52,53]. A *N*_eq_ value of 1 means total rigidity, 4 is flexible, 6 is highly flexible, and 8 or more is a disordered region [54].

As for RMSF, CDR1 is considered to be going to a flexible conformation. The wild type has the lowest *N*_eq_ value of 3 (i.e., considered fairly flexible); all other systems see this value increase; and the extreme case is the double mutant 32G-99G which has a double value (a *N*_eq_ value of 6, i.e., highly flexible).

For CDR2, it is always highly flexible (with a common maximum *N*_eq_ value of 5). For all systems, as for RMSF, the beginning of FR3 (positions 58 to 63) is associated with a flexible *N*_eq_ value of 4. This FR is like most FRs strongly composed of β-strand, but this one is a very small β-strand and finished with a small helical zone (that could be considered a β-turn [55,56]). This region has the greatest flexibility (as seen in other systems [57]).

CDR3 has the least flexibility with *N*_eq_ values ranging between 2 and 4. Surprisingly, although it might be expected to be more flexible due to the absence of side chains, the most rigid system is the double mutant Glycine. The use of PBs also allows us to see that FR1 is slightly deformable with a max *N*_eq_ of 2 for wild type and of 4 for 32A-C99, when it is only 1.4 for C32-99A. Those results show that the second disulfide bond has an impact mainly on CDR1 deformability. Nonetheless, the limited effect on CDR3 is rather unexpected.

### 2.4. Comparison of Local Protein Conformations

**Figure 5:**
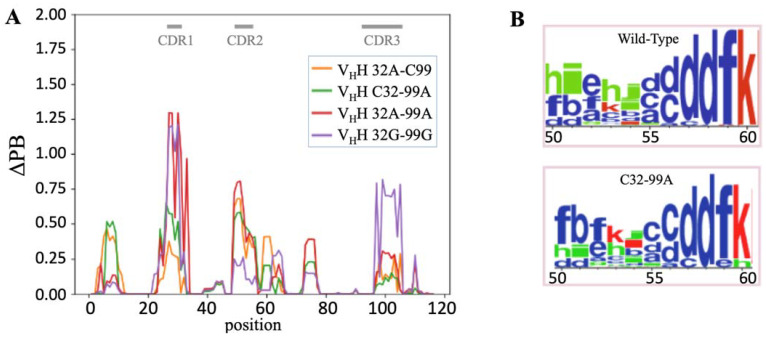
Local analyses. (**A**) ΔPB (difference in PB signature) between wild type and 32A-C99 (orange), C32-99A (green), 32A-99A (red), and 32G-99G (purple). The dashes at the top of each figure in grey represent the CDR positions. An example of the difference in the PBs of the wild-type and the 32A-C99 mutant is shown in (**B**) PB WebLogo [59] between positions 50 and 60.

To deepen the analysis of the local information used, ΔPB (a measure that quantifies the differences in PB frequency) can be used [58]. Analysis of ΔPB (see Figure 5A) allows for a finer description of the conformational diversity between wild-type and mutant structures. ΔPB varies between 0, i.e., exact similar PB frequencies observed at the same position between two simulations, and 2, i.e., entirely different PB frequencies observed at the same position between two simulations.

FRs have a ΔPB value not exceeding 0.5 (one-quarter of PB occurrences are different). It is found for FR1 for 32A-C99 and C32-99A while double mutants have a wild-type-like PB signature for FR1. For FR3, few positions have ΔPB higher than 0.25 (1/8th of PB occurrence is different). In FR3, it concerns position 75, an Alanine, which is in direct contact with CDR1 N-ter. Hence, the loss of the disulphide bridge has a limited impact on FRs. The only unexpected case is found for the N-ter (position 8) of FR1 that is far from CDRs.

As expected, the ΔPB is higher for the CDRs. A first interesting point is that each system is particular and that single and double mutants do not have the same behaviours at all. The second point is that the ΔPB values are quite different for each CDR.

For CDR1, the maximal ΔPB values are 1.25 for both double mutants (32A-99A and 32G-99G), i.e., PB distinct distribution (65%), while they reach 0.60 for C32-99A and 0.30 for 32A-C99. The maximal ΔPB value for CDR2 is 0.75 for 32A-99A, 0.60 for 32A-C99, 0.50 for C32-99A, and only 0.25 for 32G-99G. For CDR3, it is again totally different, with a maximal ΔPB value for 32G-99G (0.75), while all the other systems have ΔPB values less than 0.25.

Every mutant system is not equivalent. Hence, C32-99A had maximal ΔPB values of 0.50 for FR1, CDR1, and CDR2 and 0.15 for CDR3. Its symmetrical 32A-C99 had similar slightly lower values (0.4 for FR1, 0.3 for CDR1, 0.6 for CDR2, and 0.25 for CDR3), making these two systems the least impacted. For the double mutants, the results were quite different, with a low impact on FR1 (maximal ΔPB values of 0.2 and 0.05, resp.), a strong impact on CDR 1 (1.25 for both), and completely opposite behaviour on CDR2 (0.75 and 0.20, resp.) and CDR3 (0.25 and 0.75, resp.). These results show that the number of mutations and the type of change have a direct impact on local conformations. Nonetheless, these effects do not add up.

Thus, we saw that a maximum of one-third of the local conformations were sampled differently for CDR2 and CDR3, and two-thirds were sampled differently for CDR1. This could seem important at first glance, but actually, the problem is more complex. Indeed, Figure 5B shows PB distribution with WebLogo representation for positions spanning from 50 to 60 (i.e., CDR2). They are associated with ΔPB around 0.65, i.e., one-third of difference. However, no new PB appears. It is always the same PBs in one or another system, but with a clear change in frequencies. For instance, at positions 50–54, the majority PB series is *hieid* for the wild type while it is PB series *fbfklc* for 32A-C99 at the same positions. Hence, for each system, both PB word series are found but with a frequency inversion of the major word. This example is representative of all the CDRs behaviours. We have (i) a restricted number of protein local conformations, but (ii) mutation(s) does not make a new local conformation, only a change(s) in the conformer’s occurrence, i.e., the difference in ΔPB. This analysis allows for a precise comparison of V_H_H structures that can be considered similar but with small dynamic differences despite sequence differences. By consequence, it explains why the binding could remain similar regardless of the presence or absence of the second disulphide bridge.

## 3. Discussion

Analysis of V_H_Hs has revealed the presence of an additional disulphide bridge between CDR1 and CDR3 (see Figure 2) [36,37,41]. They can be seen in 25% of all V_H_Hs [13,42,43]. Different experiments underline that it was not as essential as expected for the specific properties of V_H_Hs [44,45,46], and this V_H_H is an excellent representative of all these types of VHHs with an extra disulphide bridge. In order to understand its influence, a molecular dynamics study has been set up and analysed. MDs have shown its interest in analysing V_H_H behaviours [43,57,60,61,62,63,64,65,66,67,68,69,70,71,72,73,74].

Long simulations followed by detailed analysis of the systems studied were carried out to achieve this. A variety of mutations have been used to consider the absence of the extra disulphide bridge, and the results of the analyses of these mutant systems are complex. Thus, single mutants behave more like the wild type than double mutants. Using classical approaches (i.e., RSMF), this study shows that the absence of the second disulphide bridge appears to destabilize the local conformations of V_H_H. Nonetheless, further information can be obtained by analysing the ΔPBs.

All the analyses show the impact of the second disulfide bond’s absence on the rigidity/flexibility of the three CDRs. A contrario, it is highly limited on CDR1 and CDR3, mainly changing some equilibrium between conformers. Thus, it appears that breaking the disulphide bridge does not significantly influence V_H_H conformation. Interestingly, an impact on the CDR2 rigidification has been identified, i.e., an increase in deformability is also observed but with no real global consequences.

Hence, there is no exploration of new conformations but a change in the equilibrium between the different observed conformations. This explains why the interaction of V_H_Hs with or without this second bridge is not really affected, but their rigidity is slightly reduced. Therefore, the additional disulphide bridge does not seem to be an essential part.

Thus, this additional disulfide bond seems to not play a major role in the question of local and global conformations and just has a subtle impact on the occurrence of the observed conformers. A slight decrease in the thermodynamic stability of V_H_H domains is often observed. The question remains as to the possible influence of the disulfide bond on the protein folding process, i.e., its kinetic effects. Recent studies confirmed that the thermal stability of V_H_H actually reflects thermodynamic stabilities in a wide range of temperatures [75,76], as previously stated [77,78,79]. Since almost a quarter of V_H_Hs have this additional disulphide bond, it seems logical that this property should not be strongly affected, but it would be good to have experimental data, which are quite difficult to obtain.

Also, the thermal stability and global thermodynamic equilibrium of different V_H_Hs can be explained by a higher folding stability to all temperatures. This property is particularly true in working conditions of around 37 °C for a therapeutic V_H_H. This work therefore allows us to see that this second disulfide bridge, present or absent, will not have too important consequences for the design of therapeutic V_H_Hs.

## 4. Materials and Methods

### 4.1. V_H_H Structure

The structure of one representative V_H_H with two disulphide bridges was downloaded from the Protein Data Bank website (https://www.rcsb.org, accessed 19 December 2023) [80], with PDB id 1YC7 [35]. Chain A was selected, with only the first residue not resolved. The position of the classical first disulphide bridge is between residues 22 and 95, and the position of the second disulphide bridge is between residues 32 and 99. The structure was analysed by classical approaches such as MolProbity [81] and visually with PyMOL 2.4.0 software (https://pymol.org/2/, accessed 29 August 2023) [10,11,12].

### 4.2. Molecular Dynamics

Molecular dynamics (MDs) simulations were performed using GROMACS 2021.4 software [82] with the CHARMM-36 force field [83], considering five different systems: (i) the initial PDB structure of the V_H_H, i.e., C32-C99, (ii) the first single mutant with change in one Cysteine by an Alanine, i.e., C32A-C99 (for simplification, it will be noted 32A-C99), (iii) the second single mutant with change in the second Cysteine by an Alanine, i.e., C32-C99A (it will be noted C32-99A), (iv) the first double mutant with change in the two Cysteines by two Alanines, i.e., 32A-99A, and (v) the first double mutant with change in the two Cysteines by two Glycines, i.e., 32G-99G. Each structural system was energy-minimized for 500 steps of steepest descent and 500 steps of conjugate gradient using GROMACS suite. The V_H_H structures were soaked in a rhombic dodecahedral simulation box with TIP3P water molecules. After that, charge neutralization was achieved by adding sodium and chloride ions with one atmosphere of pressure and 310 K of temperature to correspond with the experimental conditions.

The MDs protocol is standardized through our previous works [58,60,84]. After 1 nanosecond (nsec) of equilibration, each system was simulated through multiple classical independent production runs with 4 replicates of 250 nanoseconds as in [59]. The equilibration protocol consists of one step with an NVT system and three more with an NPT system (with position restraints on protein atoms). During the first step in NVT and the second step in NPT, the protein is totally constrained and unable to move, while the equilibration affects the water molecules. In the third and fourth step in NPT, the constraints on the protein are slowly released. Molecular conformations were saved every 100 picoseconds for downstream analysis. This produced 1 µs of MDs simulation for each system.

Trajectory analyses were carried out with the GROMACS software, in-house Python, and R scripts. Root mean square deviations (RMSDs) and root mean square fluctuations (RMSFs) were calculated on Cα atoms only.

### 4.3. MDs Analysis

The analysis of MDs was performed using classic tools, such as RMSD and RMSF, and other more innovative ones such as PBxplore [51], a tool developed within the team to analyse Protein Blocks throughout the MDs simulation. RMSD (root mean square deviation) quantifies structural variations during dynamics by comparing each frame to a reference structure; here, this is the starting frame. For each frame, an average of the differences between the reference positions and the positions of the current frame is taken in order to have an RMSD value per unit of time.

The RMSF (root mean square fluctuation) is similar to the RMSD, by determining the fluctuation of each residue following the same principle as for the RMSD, i.e., a comparison with a reference. But this time, it is the average position of each residue, calculated during the simulation time, and thus the measure of the difference between the current position and the average position in order to have a flexibility value per position.

The assignment of secondary structures was carried out using the Dictionary of Secondary Structure of Protein (DSSP) [85,86]. The DSSP provides eight states of description (α-helix, π-helix, 3_10_-helix, β-strands, β-turns, bents, β-bridge, and coil). Thus, from the trajectory file generated by GROMACS, the DSSP assigns the secondary structure element for each time interval. This analysis allows us to easily see the stability or not of the protein secondary structure elements as a function of time.

Protein Blocks (PBs) are a structural alphabet composed of 16 local prototypes [50,87,88]. Each specific PB is characterized by the φ, and ψ dihedral angles of five consecutive residues with each PB assignment are focused on the central residue. Obtained through an unsupervised training approach and performed on a representative non-redundant databank, PBs give a reasonable approximation of all local protein 3D structures [89]. PBs are very efficient at tasks such as protein superimpositions [90,91] and MDs analyses [54,92,93,94]. They are labelled from *a* to *p*: PBs *m* and *d* can be roughly described as prototypes for α-helix and central β-strand, respectively. PBs *a* to *c* primarily represent β-strand N-caps and PBs *e* and *f* representing β-strand C-caps; PBs *a* to *j* are specific to coils, PBs *k* and *l* are specific to α-helix N-caps, while PBs *n* to *p* are specific to α-helix C-caps. PB assignment was carried out using our PBxplore tool (https://github.com/pierrepo/PBxplore, accessed 19 May 2024) [51].

PB assignments are carried out for each residue of the C-domain and over every snapshot extracted from MDs simulations. The equivalent number of PBs (*N_eq_*) is a statistical measurement similar to entropy that represents the average number of PBs for a residue at a given position. *N_eq_* is calculated as follows [50]:

$$N_{eq}=exp\left(-\sum_{x=1}^{16}f_{x}lnf_{x}\right)$$

where *f_x_* is the probability of PB *x*. A *N_eq_* value of 1 indicates that only one type of PB is observed, while a value of 16 is equivalent to a random distribution. To underline the main differences between one system and another one for each position, the absolute difference Δ*N_eq_* between corresponding *N_eqs_* values was computed.

However, since the same Δ*N_eq_* value can be obtained with different types of blocks in similar proportions, we have defined a complementary measure, ΔPB, that evaluates a change in PB profile by calculating the absolute sum of the differences for each PB between the probabilities of a PB *x* to be present in the first and the second forms (*x* goes from PB *a* to PB *p*). ΔPB is calculated as follows [59]:

$$∆PB=\sum_{x=1}^{16}\left|\left(f_{x}^{1}-f_{x}^{2}\right)\right|$$

where $f_{x}^{1}$ and $f_{x}^{2}$ are the percentages of the occurrence of a PB *x* in the first and the second system, respectively. A value of 0 indicates perfect PBs identity between the 1st and 2nd systems, while a score of 2 indicates a maximum total difference.

PBxplore also uses WebLogo to provide a dedicated PB logo output [59].

## Data Availability

Molecular dynamics trajectories are available on request.
